# Editorial: Platelet and megakaryocyte dysfunctions in infectious diseases

**DOI:** 10.3389/fimmu.2023.1175200

**Published:** 2023-03-28

**Authors:** Alice Assinger, Claude Capron, Fernando Real, Morgane Bomsel

**Affiliations:** ^1^ Department of Vascular Biology and Thrombosis Research, Center for Physiology and Pharmacology, Medical University of Vienna, Vienna, Austria; ^2^ Service d’Hématologie Hôpital Ambroise Paré (AP-HP), Boulogne-Billancourt, France; ^3^ Université de Versailles-St Quentin en Yvelines, Versailles, France; ^4^ Laboratory of Mucosal Entry of HIV and Mucosal Immunity, Institut Cochin, Paris, France; ^5^ INSERM U1016, CNRS UMR8104, Université de Paris Cité, Paris, France

**Keywords:** platelets, thrombosis, bleeding, infection, viruses, bacteria, COVID-19

Platelets constantly patrol the vasculature to prevent blood loss upon injury in a process termed haemostasis. Upon rapid adhesion to sub-endothelial matrixes, platelets become activated and recruit further platelets to form aggregates which, after stabilizaton by the fibrin mesh generated *via* the coagulation system, ultimately seal the injured vessels. Apart from their central role in haemostasis, platelets also regulate vascular integrity as well as tissue repair mechanisms and are crucially involved in immune-regulation. It is therefore not surprising that platelets play an important role in infectious diseases.

More evidence emerges as to their unexpected and experimentally poorly explored roles. On the edge between beneficial or harmful activities, platelets are capable of harbouring infectious viral and non-viral pathogens, therefore acting as Trojan horses for pathogen dissemination. They can directly participate and actively interfere with immune responses to these pathogens, allowing antigen presentation or serve as shelters for these pathogens to protect them against immune surveillance. Platelets are thus an invaluable physiological cellular model for fundamental research on host-pathogen interaction such as antigen presentation, pathogen endocytosis, and exocytosis of virulence factors or inflammatory mediators, as well as cell-to-cell transfer of microorganisms.

This special Research Topic issue unites different aspects of platelets and megakaryocytes in infection. It explores how they are targeted, subverted, exploited or functionally modulated by pathogens, directly or indirectly, in their battle to establish infection and multiply host cell organisms. The role of platelets and their progenitors remains poorly understood. Recent discoveries have challenged the traditional concept of platelets as a simple haemostatic cell.

In their review Tokarz-Deptula et al. summarized the current knowledge on the burning question of what precise function platelets play in bacterial and viral infections, with a special focus on the endovascular environment. They described how platelets interact and contribute to the damaged endothelium as well as exploring their role in the immune system.

Review Li et al. discussed the bidirectional interactions between platelets and various microbial pathogens and how this impacts innate and adaptive immune responses. The authors nicely explore how platelets are involved in early detection of invading pathogens and are actively recruited to sites of infection. Platelets exert direct antimicrobial effects to eliminate or restrict dissemination, or shape the host immune response. As such, this review provides important insights on how microbial pathogens developed strategies to alter platelet count and functions.

Platelets not only interact with bacteria but also play a role in platelet-viral interactions. Schrottmaier et al. discussed the multifaceted and complex causes of platelet dysfunction in viral infections. While some viruses directly interact with platelets and/or megakaryocytes to modulate their functions, immune and inflammatory responses also directly and indirectly favour platelet activation. This interplay explains the complex mechanisms whereby platelet activation results in increased platelet consumption and degradation, which in turn contributes to thrombocytopenia in these patients. The authors further hypothesise that platelets have a bi-phasic role in viral infection, in which initial platelet hyper-activation is followed by platelet exhaustion/hypo-responsiveness. This would explain why infections increase both the thrombotic and bleeding risks in patients.

In the review by Xiang et al. summarized the current knowledge of platelet-mediated effects on pulmonary microcirculation during the onset and progression of COVID-19. Platelets become activated and consumed during COVID-19 and low platelet counts are associated with disease severity. Indeed, activated platelets fail to protect vascular integrity, leading, in turn, to increased permeability. The authors discussed how the procoagulant surface of activated platelets fosters thrombus formation and thereby contributes to pulmonary thrombotic complications. The benefits of current antiplatelet and anticoagulation therapies are also discussed for COVID-19 associated dysregulation of pulmonary microcirculation.

In addition to viral haemorrhagic fever and SARS-CoV-2, other viral infections also modulate platelet functions. In this special Research Topic issue, Azevedo-Quintanilha et al. explored the role of platelets in Chikungunya fever. Chikungunya infection, a viral disease transmitted by mosquitoes, leads to fever with joint pain and swelling, which, in rare cases, persist for months. In a prospective study on over one hundred patients with Chikungunya infection, the authors demonstrated that patients show increased platelet activation with increased platelet-inflammasome engagement during Chikungunya infection. In *in vitro* studies, they confirmed direct activation of platelets by Chikungunya virus, suggesting involvement of platelets in immune processes leading to accelerated inflammatory responses in these patients.

Even less explored is the mother cell of platelets, the megakaryocyte, a unique and poorly studies cell that is polyploidal and gigantic in size. Its ability to migrate out of the bone marrow and to produce platelets locally is also the subject of recent and remarkable discoveries including their implication in infectious respiratory diseases like COVID-19.

A review by Gelon et al. summarized the current knowledge on this recently discovered phenomena. While platelets are derived from megakaryocytes in the bone marrow, megakaryocytes can also migrate to the lungs, where they get trapped in the lung capillaries and give rise to lung-borne platelets. In contrast to their bone marrow counterparts, platelet-producing megakaryocytes in the lung present specific immune signatures with functional consequences. In infectious diseases like COVID-19, this lung-resident megakaryocyte population, as well as megakaryocytes recovered in the peripheral circulation, increases tremendously in number, suggesting that these cells contribute to lung infection and immunothrombosis.

Frydman et al. went on to explore the role of these circulating megakaryocytes in sepsis. First, the authors demonstrated that, in septic patients, megakaryocytes are not only detected in the bone marrow, lungs and kidney, but are also found in the peripheral blood of patients where they could represent a new marker of infection. In *in vitro* studies, the authors further showed that upon interaction with bacteria, circulating megakaryocytes exert innate immune functions. As such, this study provided further evidence that both platelets and their progenitors, megakaryocytes, respond to microbial pathogens: megakaryocytes do so by modulating their numbers and by developing innate immune strategies to fight invading microbes.

Together, the contributions to this Research Topic deepen our understanding, as yet, unexplored, functions of platelets as well as opening new avenues of research for the treatment of infectious diseases. As pandemics and concomitant haemostatic dysregulation will remain a recurrent threat, understanding the role of platelets in viral infections represents a timely and pivotal challenge. It is only by fully understanding the multifaceted role of megakaryocyte and platelets in infections that we will be able to design new treatment strategies. Indeed, current therapeutic options for these patients are limited and new approaches are urgently needed to prevent adverse outcome.

## Author contributions

AA and MB drafted the editorial, AA, CC, FR and MB organized, revised, and edited the manuscript and agree to its style and content in its final form.

